# Supplementary figures and images for: Spatially Explicit Models to Investigate Geographic Patterns in the Distribution of Forensic STRs: Application to the North-Eastern Mediterranean
Source: PLoS One. 2016 Nov 29;11(11):e0167065. doi: 10.1371/journal.pone.0167065 (PMC5127579; doi:10.1371/journal.pone.0167065)

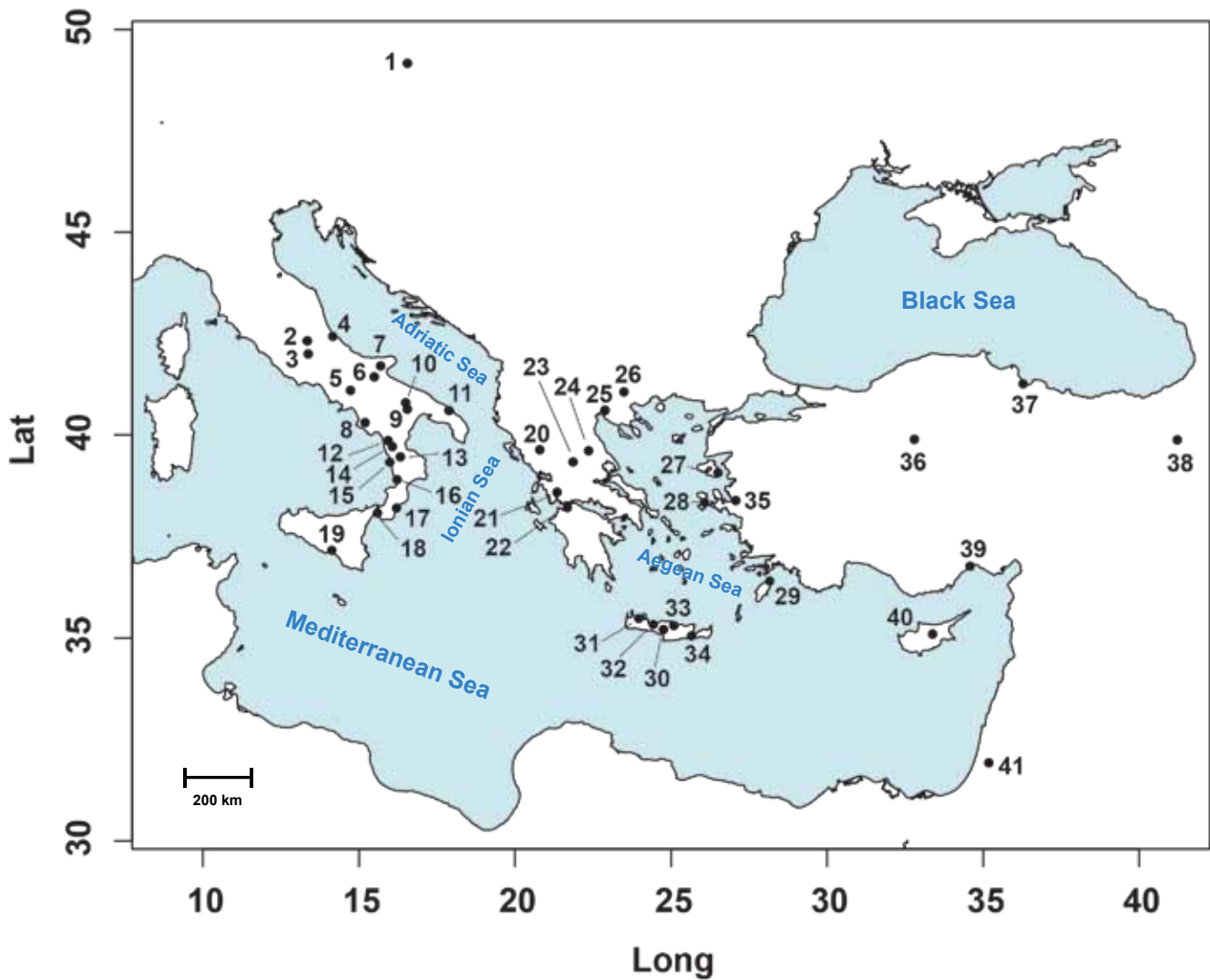

Supplement: S1 Fig — (PDF) [file pone.0167065.s001.pdf]

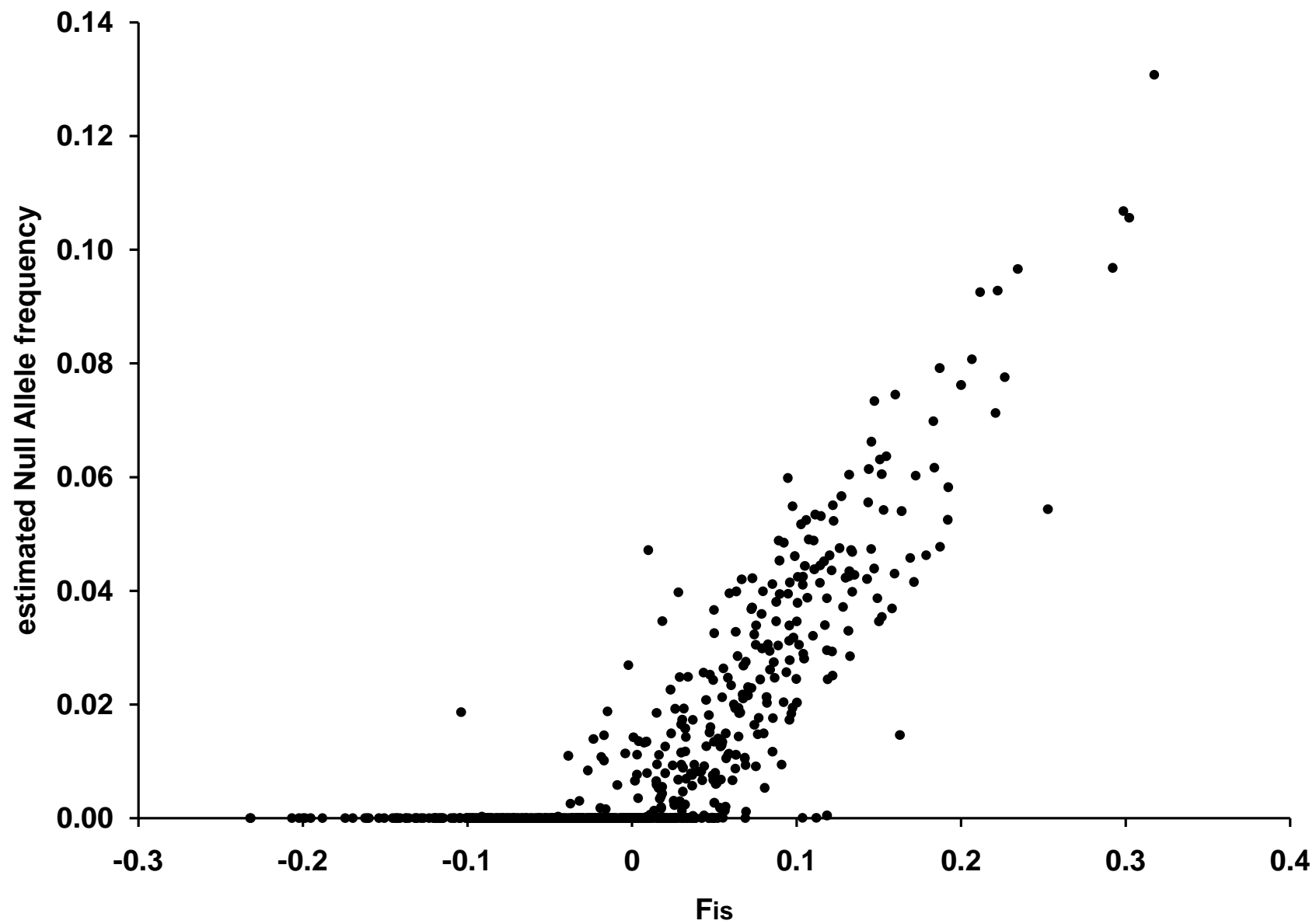

Supplement: S2 Fig — (PDF) [file pone.0167065.s002.pdf]

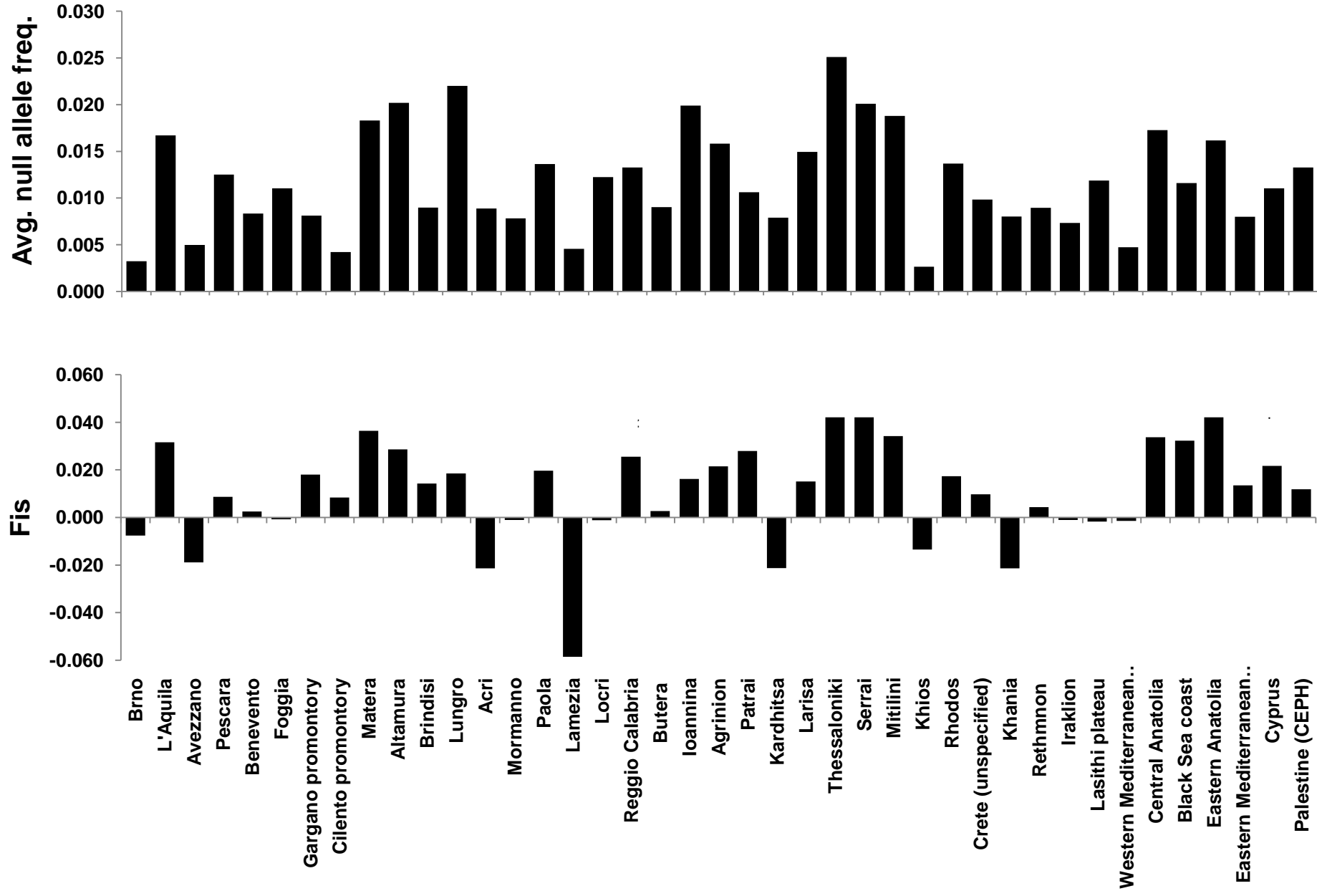

Supplement: S3 Fig — (PDF) [file pone.0167065.s003.pdf]

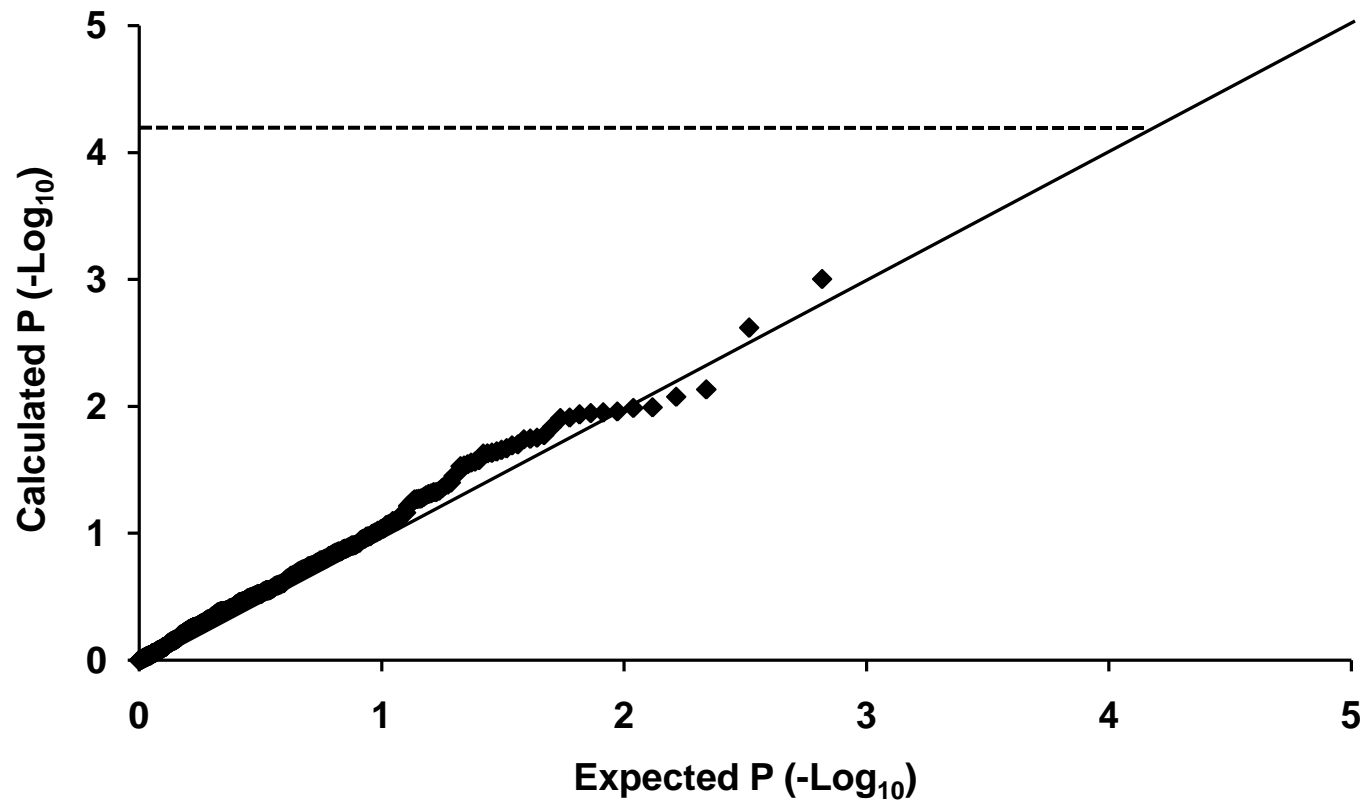

Supplement: S4 Fig — The solid line indicates identity between calculated and expected values. The dotted line represents the significance level (nominal α = 0.05) after Bonferroni correction. (PDF) [file pone.0167065.s004.pdf]

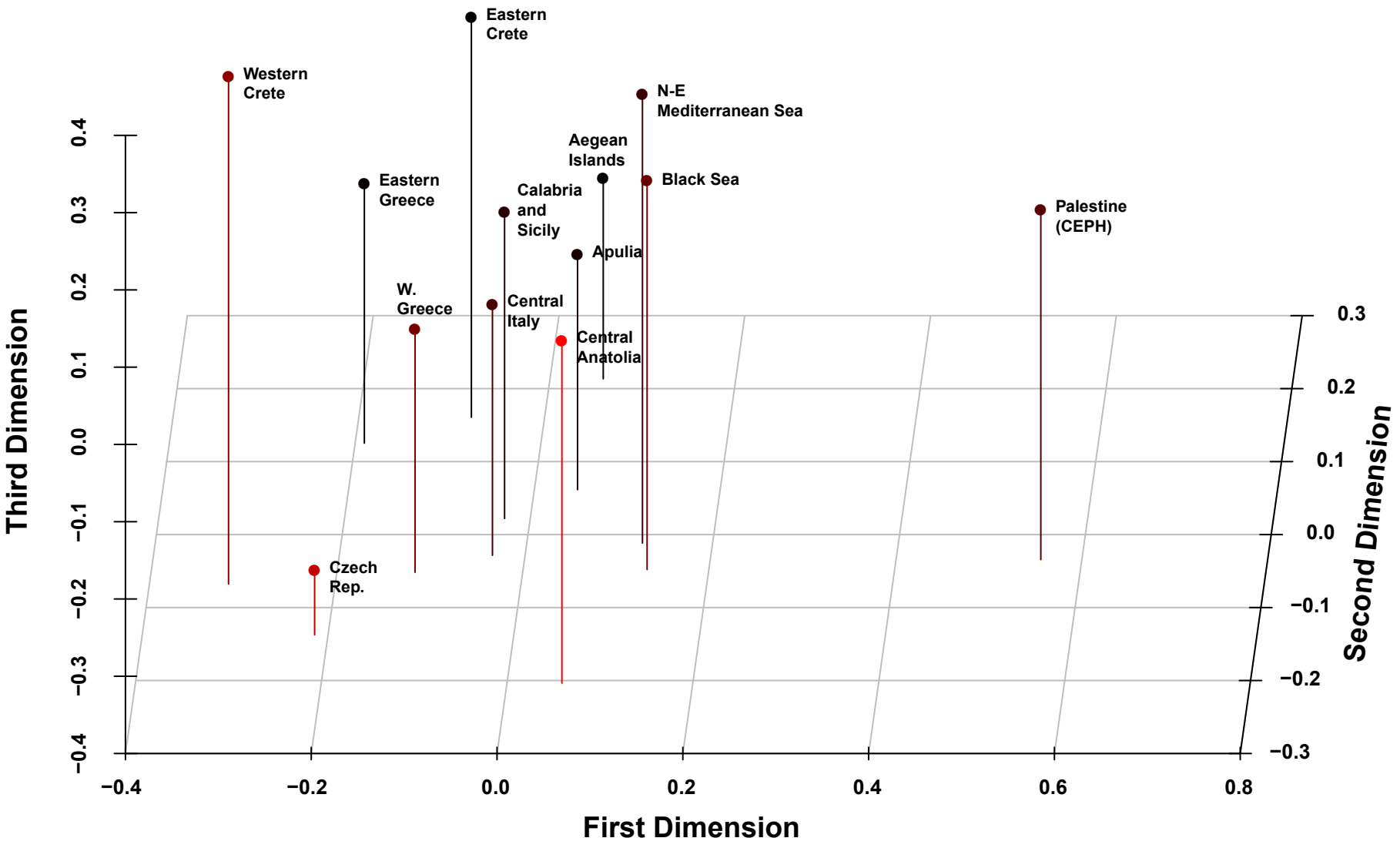

Supplement: S5 Fig — Color shades from bright red to black refer to position on dimension 2. (PDF) [file pone.0167065.s005.pdf]

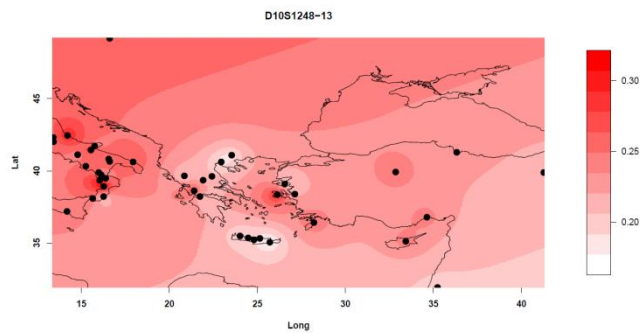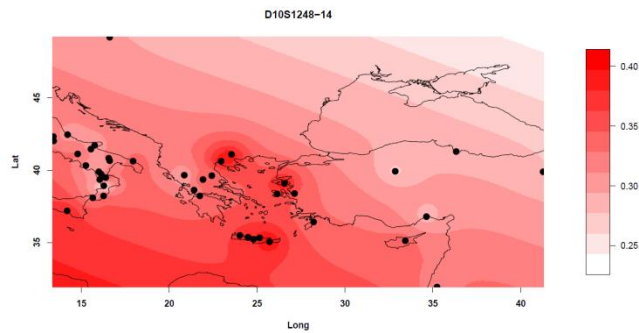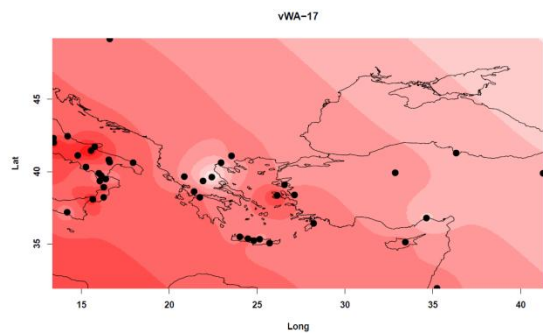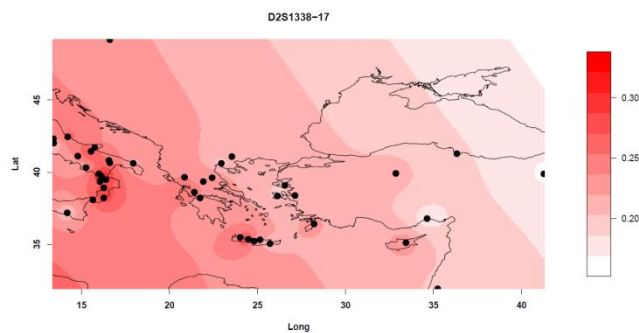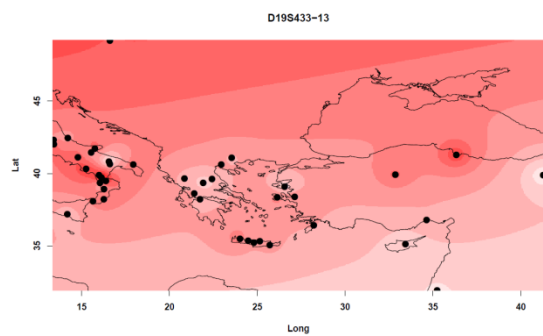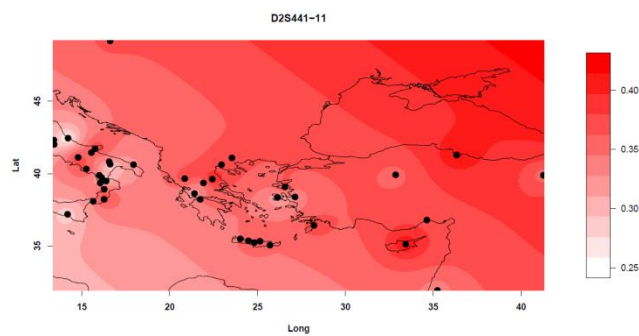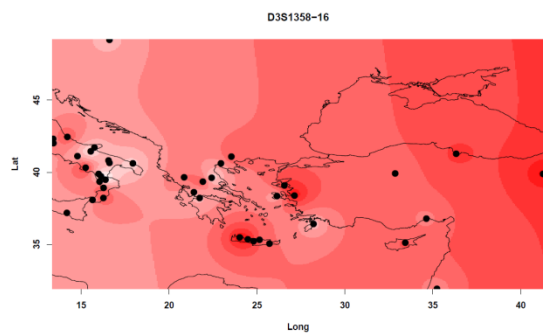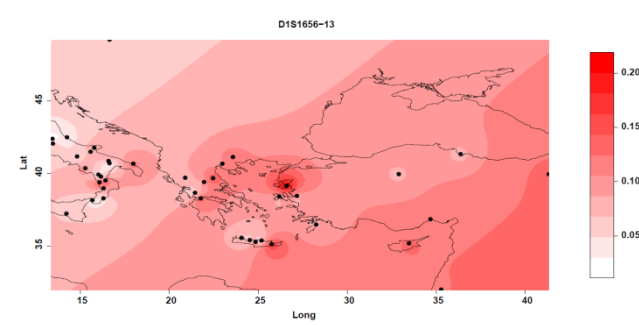

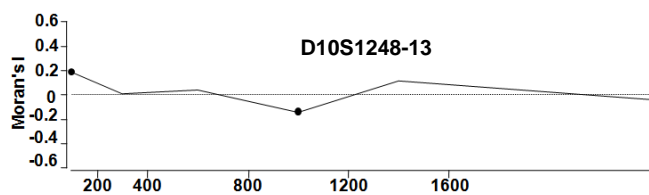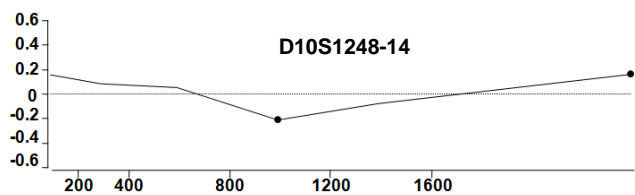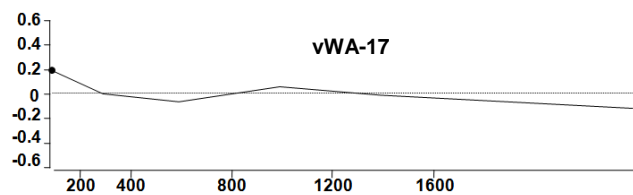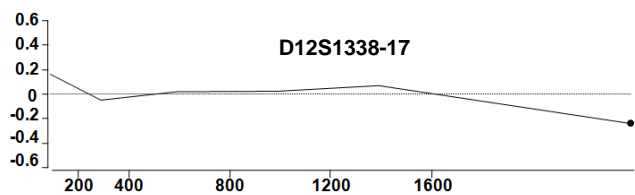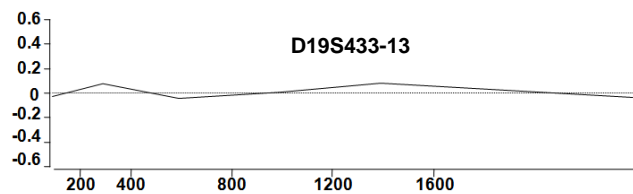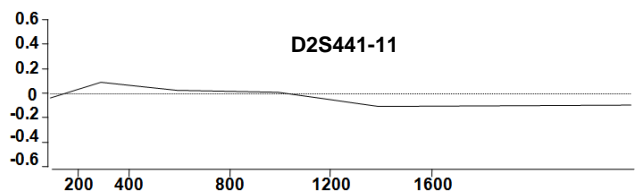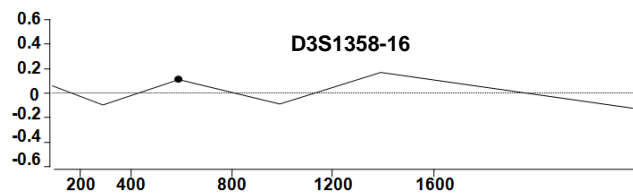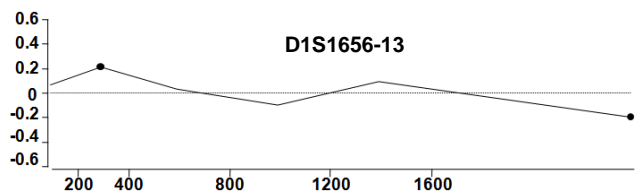

D16S539-12

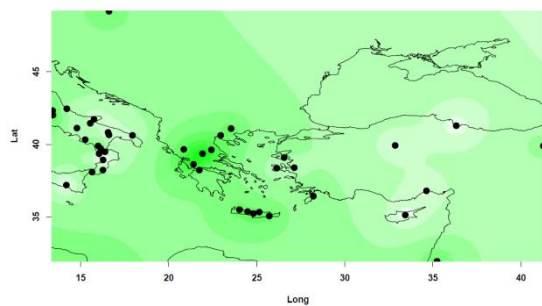

D2S1338-24

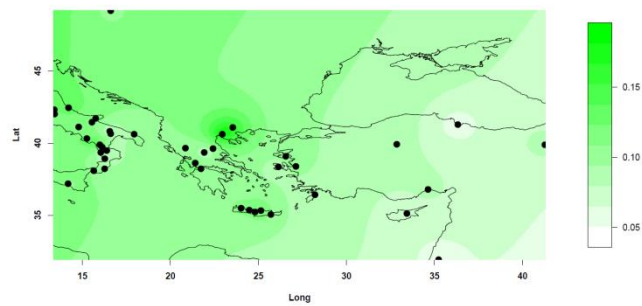

D18S51-15

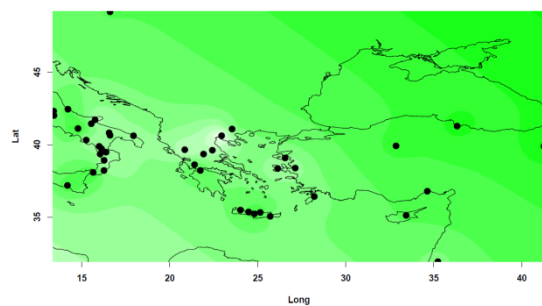

D18S51-19

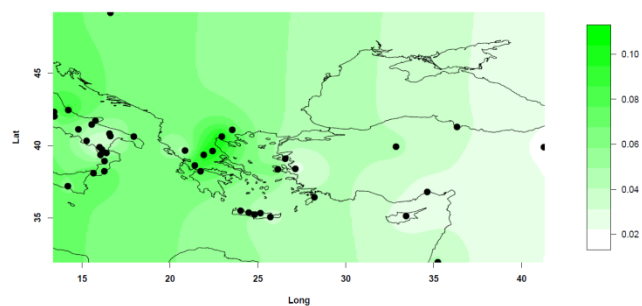

D22S1045-15

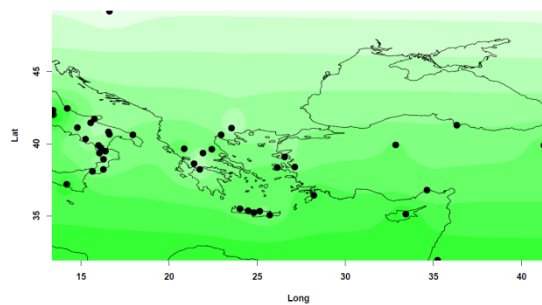

D22S1045-16

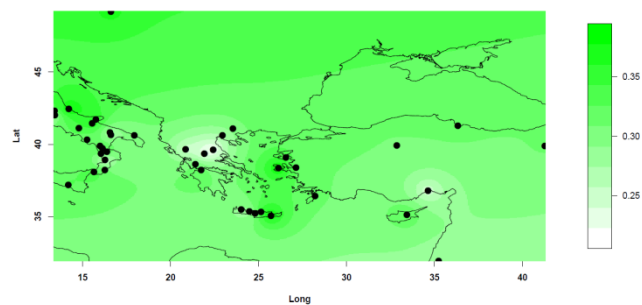

TH01-9

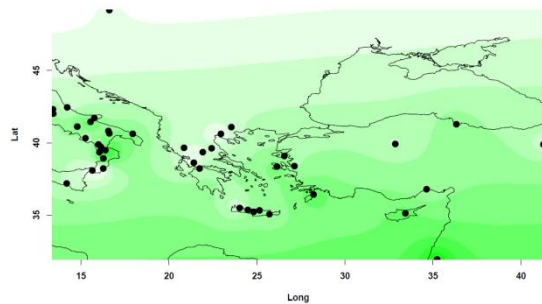

D3S1358-18

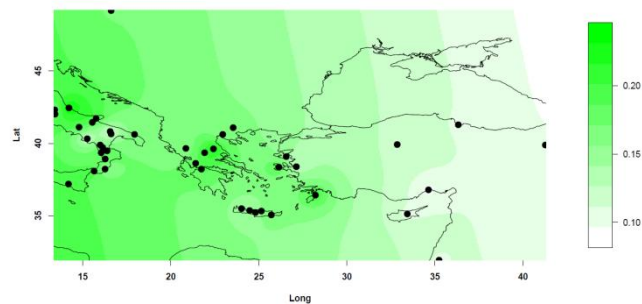

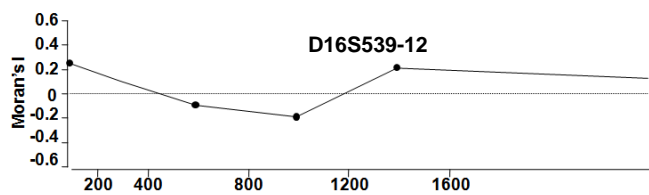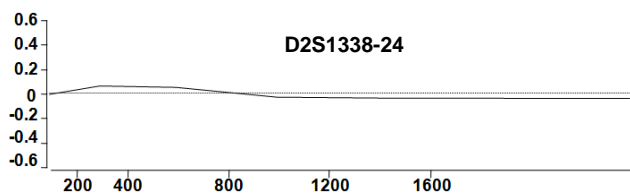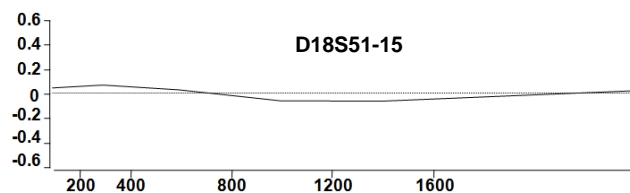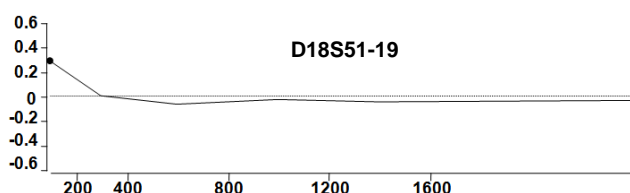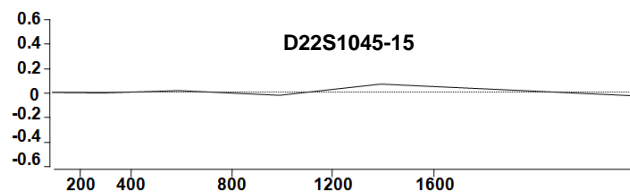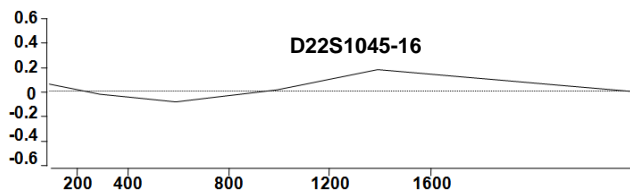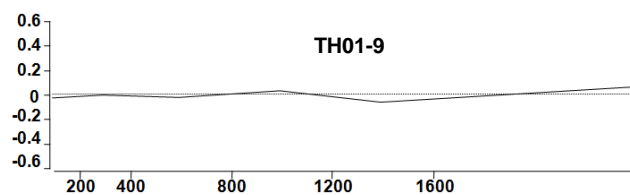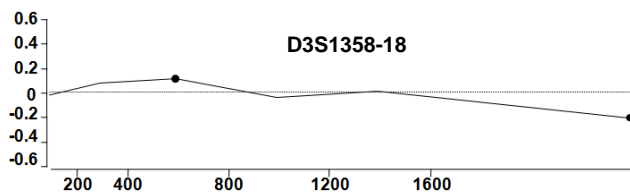

Supplement: S6 Fig — The same alleles are listed in Table 1 in the 2.5% columns. Note the different colour scale used in each map. Values outside the polygon connecting the most external points are extrapolated. For each of the two map sets, the correlograms are shown in the same order (pages 2, 4). Black dots indicate significant class-specific values, i.e. individual values for which the null hypothesis (Moran I = 0) is rejected. The global significance of the correlograms consist in checking that at least one of the coefficients retains significance after considering multiple tests for distance classes with the Bonferroni correction. Ticks on the x axis are spaced to indicate the upper bounds of distance classes. (PDF) [file pone.0167065.s006.pdf]

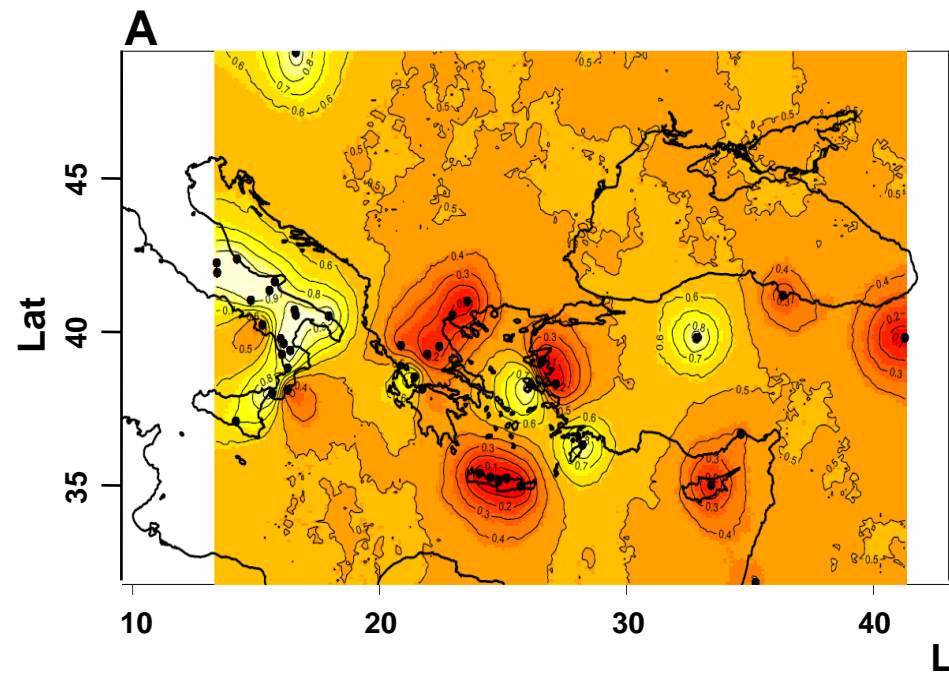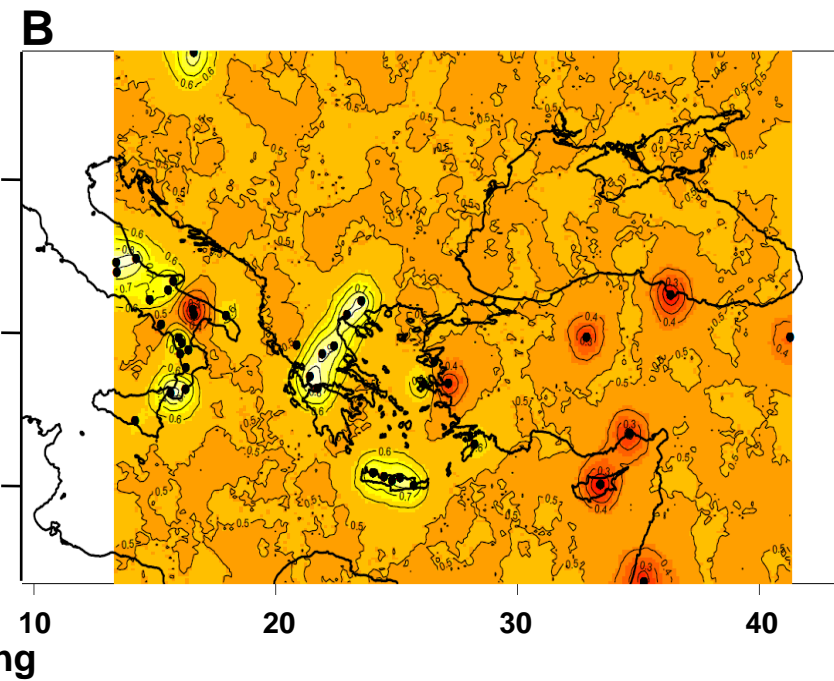

Supplement: S7 Fig — (PDF) [file pone.0167065.s007.pdf]

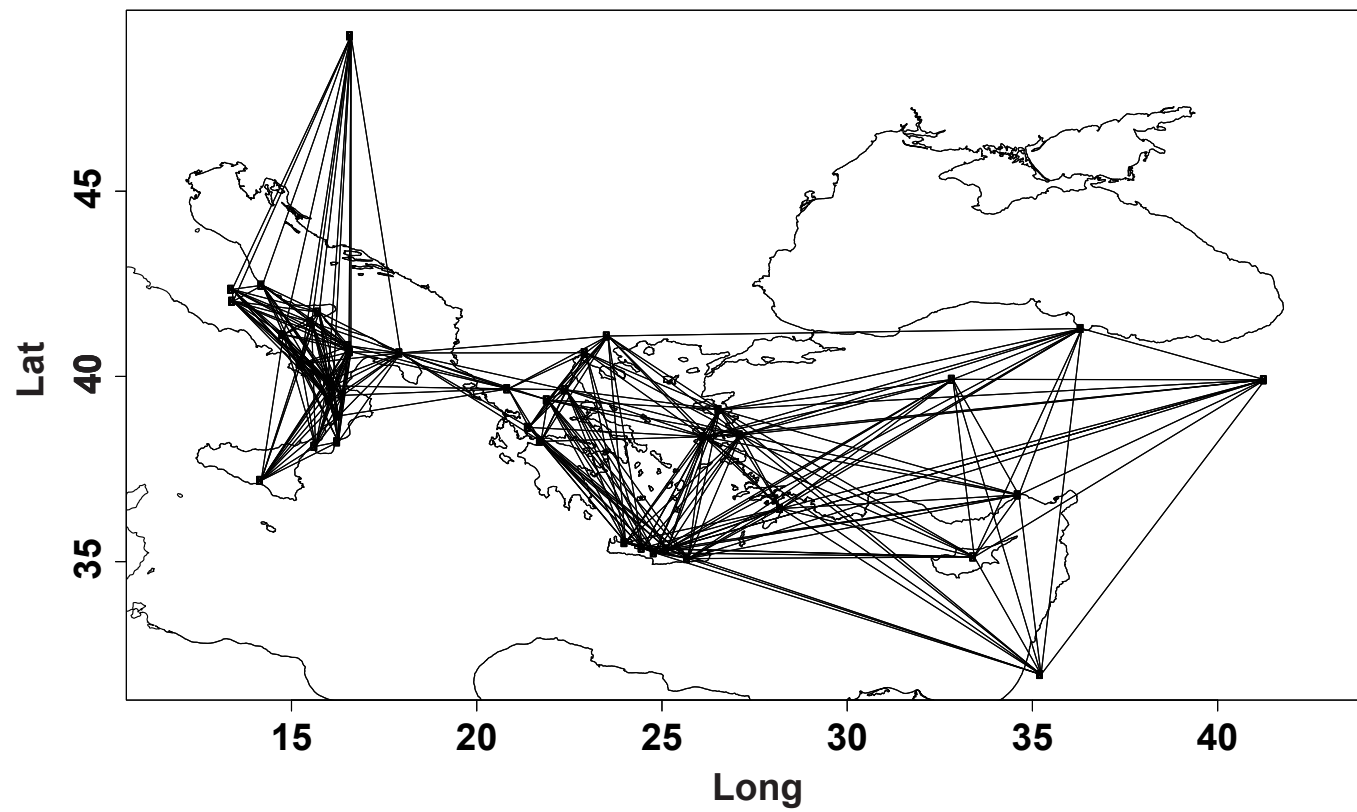

Supplement: S8 Fig — (PDF) [file pone.0167065.s008.pdf]

### Model 1

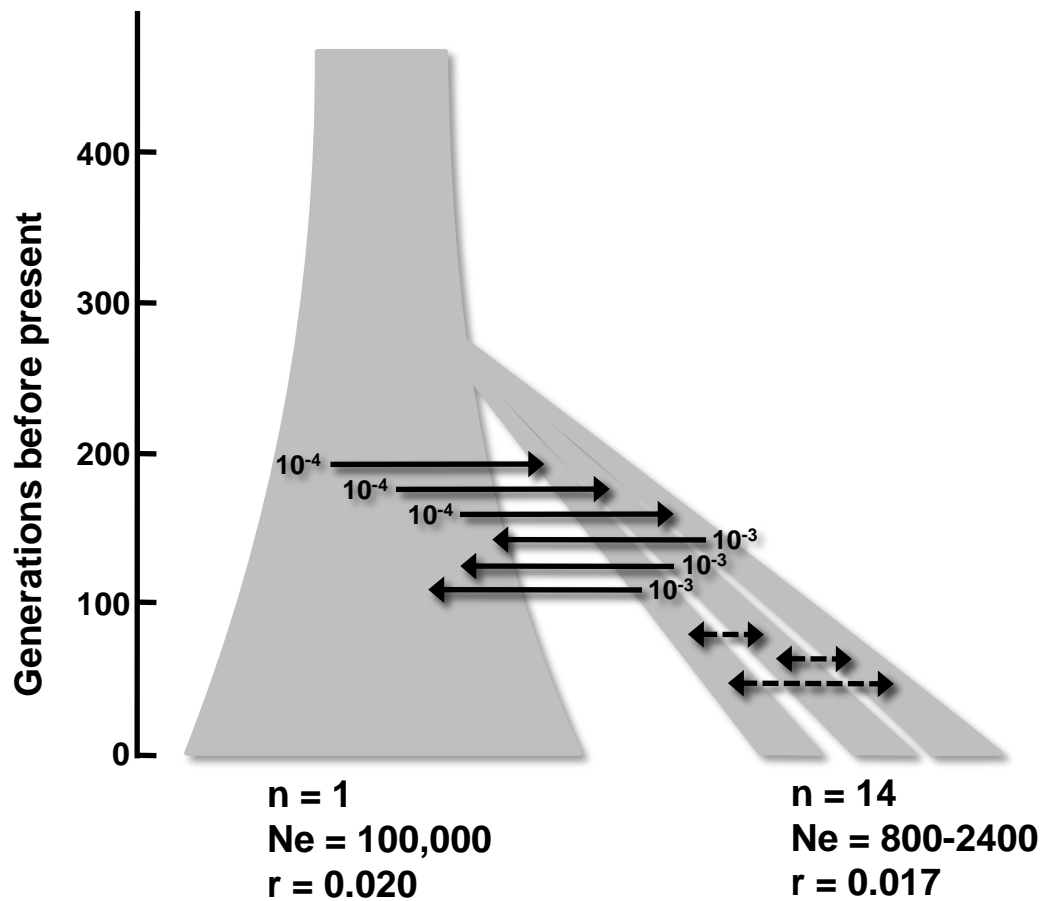

### Model 2

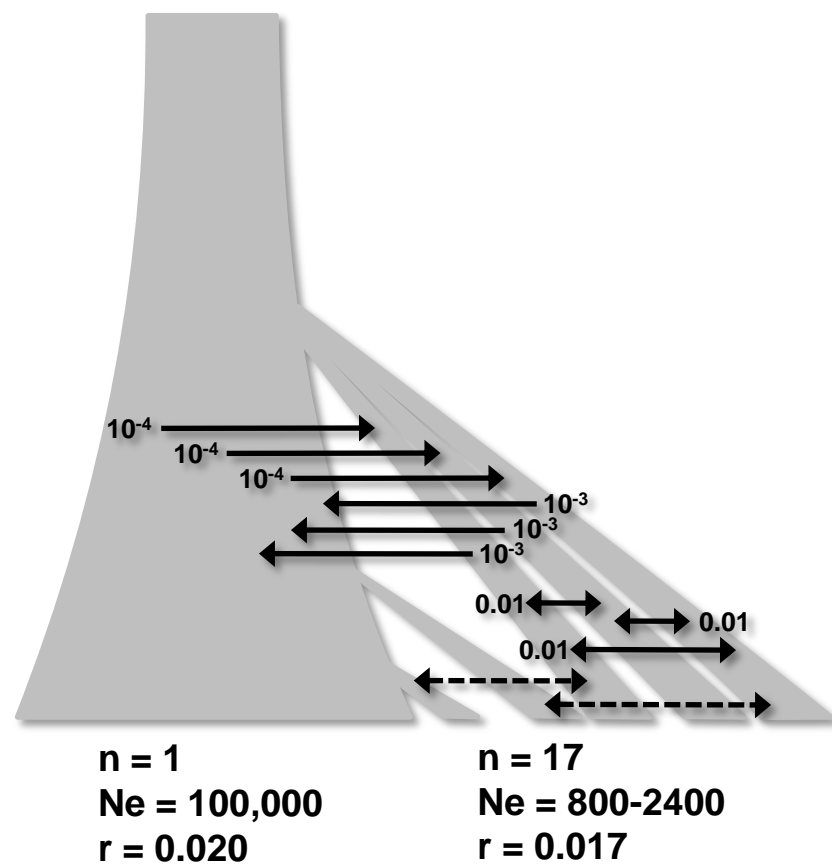

Supplement: S9 Fig — n = number of demes (only a subset shown for the sake of clarity); Ne = effective size (in gene copies); r = growth rate per generation. Black arrows represent instances of gene flow with the indicated fixed rate across simulations. Dashed arrows indicate instances of gene flow whose rate was varied across simulations. (PDF) [file pone.0167065.s009.pdf]

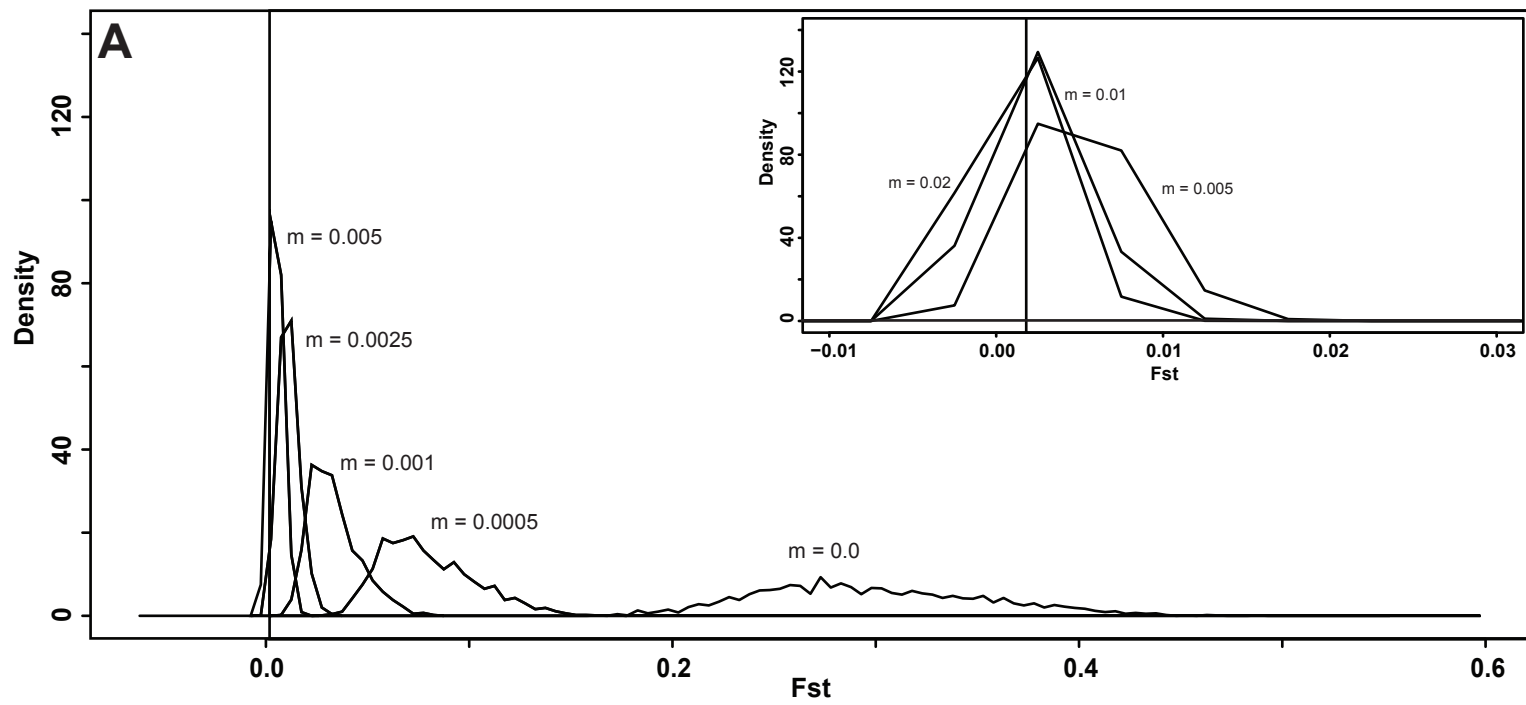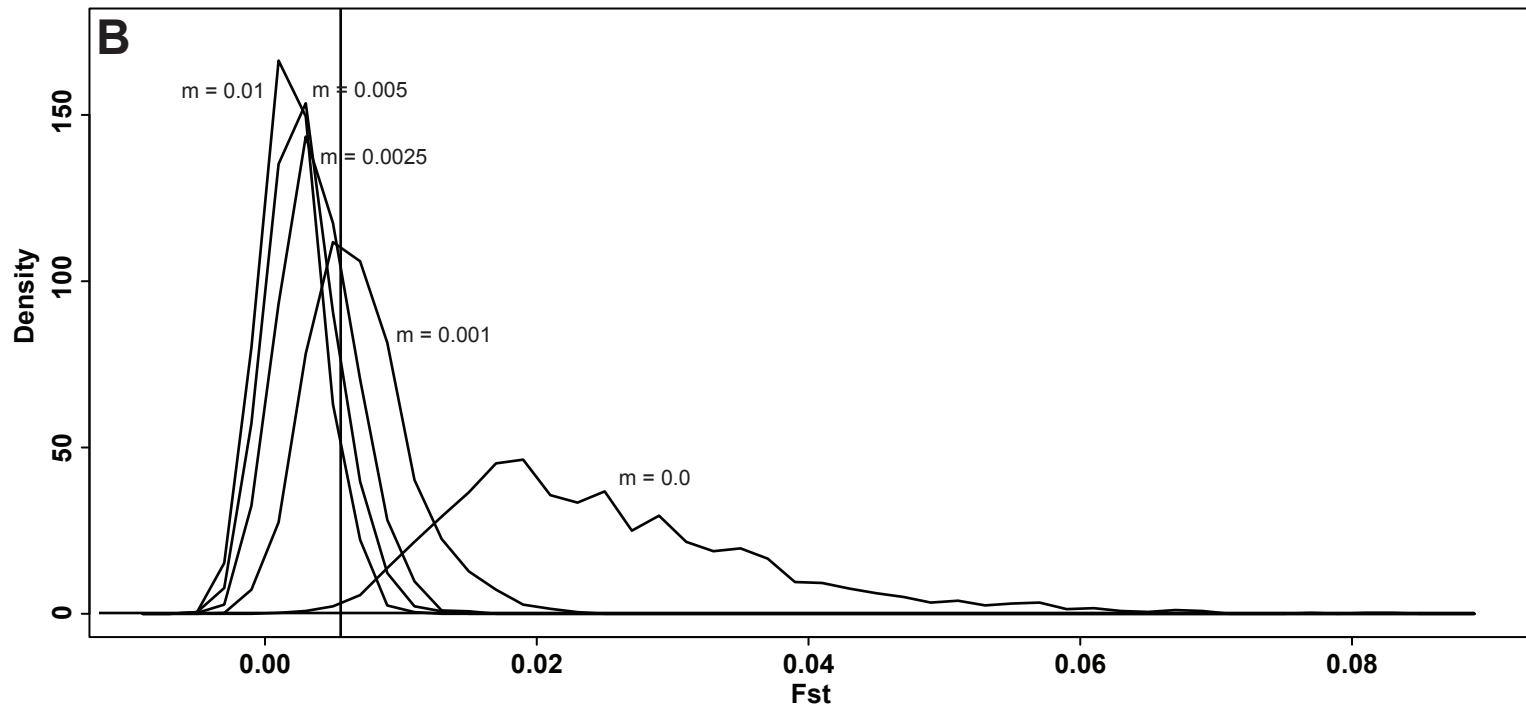

Supplement: S10 Fig — A) Model 1: migration rates (m) among the 14 demes are shown; the curves for m = 0.02, 0.01 and 0.005 are shown in the inset for clarity. B) Model 2: the numbers indicate migration rates (m) between 3 recent demes and the 14 demes of Model 2. Note the different scale of the x axis as compared to panel A. In both panels the vertical lines indicate the Fst value obtained from real data. (PDF) [file pone.0167065.s010.pdf]
