# Supplementary material for: Spatially Explicit Models to Investigate Geographic Patterns in the Distribution of Forensic STRs: Application to the North-Eastern Mediterranean
Source: PLoS One. 2016 Nov 29;11(11):e0167065. doi: 10.1371/journal.pone.0167065 (PMC5127579; doi:10.1371/journal.pone.0167065)
Supplement: S1 Text — The first one examines the effect of a 50% reduction of the sampled locations. The second and third one are based on external datasets and take into consideration forensic STRs for other samples within the same geographical frame, as well as publicly available genome-wide SNP data. (PDF) [file pone.0167065.s014.pdf]

Supplemental text

ADDITIONAL ANALYSES TO ASSAY THE ROBUSTNESS OF METHODS
(HIGH RESOLUTION MAPS AVAILABLE ON REQUEST)

Analysis of subsamples:

This analysis was aimed at evaluating the dependency of our results on the composition of the dataset and the sensitivity to the outgroups. We thus performed the spatial PCA with adegenet (Jombart et al. 2008), the identification of contributing alleles, the reduction of the dataset and the analysis with Geneland (Guillot et al. 2005) on 2 subsets of the data obtained by re-sampling and 2 based on geographic partition:

1a. A subset consisting of 50% of the locations (odd-numbered in Supplemental Table 1). This subset included also the outgroups (Czech Rep. and Palestinians);

1b. A subset consisting of 50% of the locations (even-numbered in Supplemental Table 1). This subset did not include the outgroups;

2a. A subset consisting of the 50% of the western-most locations (see Supplemental Table 1);

2b. A subset consisting of the 50% of the eastern-most locations (see Supplemental Table 1).

In order to replicate the proportion of locations connected and not connected in the network, we used a 6 nearest neighbours scheme.

Results:

Population subsets 1a,b spanned the entire geographical range as the full dataset. Accordingly, the 8 alleles with the strongest contributions to spatial PC1's obtained with these subsets had high rankings also in the list of 288 alleles of the full dataset. This was striking for subset 1b (5/8 shared alleles) , showing that the patterns captured in the full dataset truly depended on the locations at the focus of our study, and only marginally on the two distant outgroup locations.
On the other hand, alleles contributing to sPC1 when locations were partitioned on a geographic basis (2a,b), did not overlap significantly with those of the full dataset. This is not surprising, as far as geographic patterns confined to the western or eastern sectors of our range could be hardly captured by sPC1 which, by definition, weights allele frequency variance at large distances and is thus designed to highlight "global" effects. In 6 out of 8 cases the reduced datasets returned enhanced Fst's.

We next proceeded in visualizing the spatial patterns generated by the population subsets.

sPC1 and reduced datasets derived from it:

1a. The two outgroups had opposite sPC1 values (left panel). The sPC1 values clustered all Italian locations with the north-western outgroup. A single location in western Continental Greece had a null value. The grouping of Italian locations was replicated in the Bayesian clustering (right panel), but two locations in Greece and two in the Aegean Islands were also assigned to this cluster.

1b. The sPC1 values separates Italian locations from all remaining locations. The same pattern is replicated with Geneland, with the exception of locations with sPC values close to 0.

2a. The sPC1 values return a partition within Italy not observed in any of the previous analyses. This is the sole case in which the reduced dataset produced an Fst lower than the full dataset. The Bayesian clustering algorithm returned inconclusive assignment probabilities.

2b. The sPC1 partitioned locations of both sides of the Aegean Sea. The pattern was replicated with Geneland, with the exception of Rhodes.

sPC2 and reduced datasets derived from it:

1a. The two outgroups had opposite sPC2 values. Continental Greek and the West Cretan locations clustered with the North-Eastern outgroup. Two Italian locations with null sPC2 values and the West Cretan location had opposite assignments with Geneland.

1b. Two Italian locations were grouped with the Continental Grek ones. Locations with sPC2 values close to null received opposite grouping with Geneland.

2a. Many locations in Italy had very small negative or positive sPC2 values. The Bayesian clustering failed in finding two complementary clusters.

2b. sPC2 identified a cluster of Aegean locations with negative values. Geneland clustered together mainly East Crete and Mitilini.

| | Fst in full dataset | | | Fst in reduced dataset | Min-Max assignment probs. |
|---|---|---|---|---|---|
| **Pop. subset 1a** | **0.0016** | 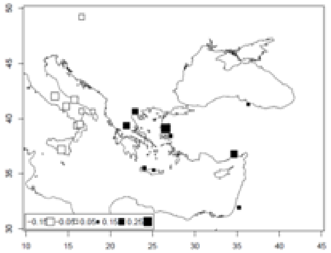 | 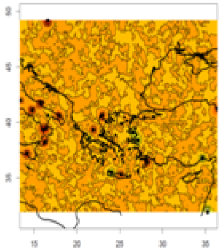 | **0.0057** | **1/0 – 0/1** |
| **Pop. subset 1b** | **0.0030** | 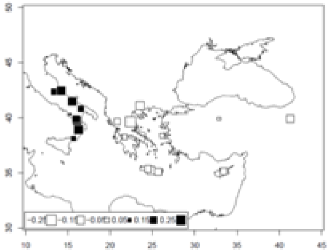 | 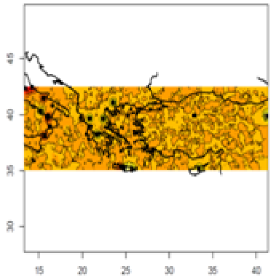 | **0.0046** | **.993/.007 - .007/.993** |
| **Pop. subset 2a** | **0.0028** | 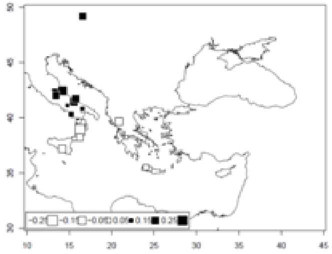 | 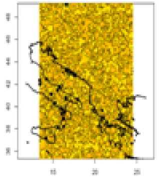 | **0.0010** | **.878/.122 - .269/.731** |
| **Pop. subset 2b** | **0.0012** | 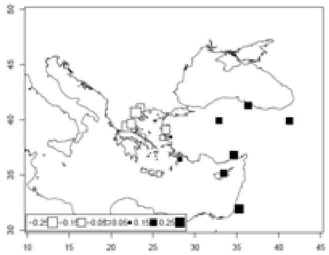 | 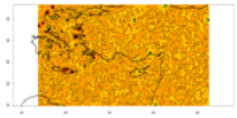 | **0.0051** | **.997/.003 – 0/1** |

**PC1**

| | Fst | | | Fst in reduced dataset | Min-Max assignment probs. |
|---|---|---|---|---|---|
| Pop. subset 1a | 0.0016 | 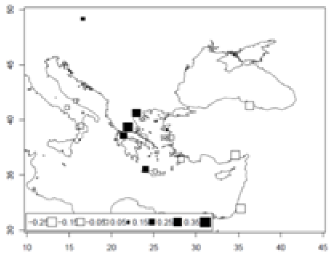 | 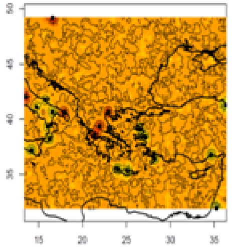 | 0.0020 | .982/.018 - 003/.997 |
| Pop. subset 1b | 0.0030 | 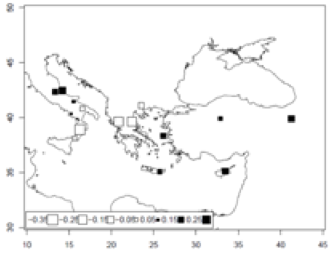 | 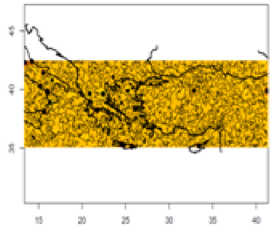 | 0.0054 | 1/0 – 0/1 |
| Pop. subset 2a | 0.0028 | 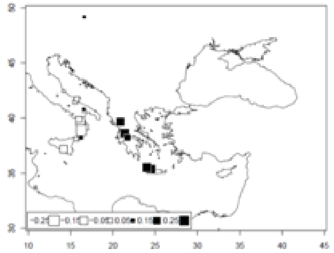 | 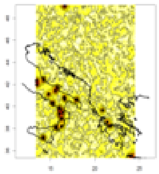 | 0.0015 | .886/.114 - .757/.243 |
| Pop. subset 2b | 0.0012 | 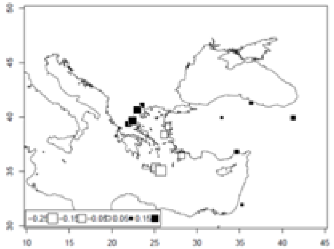 | 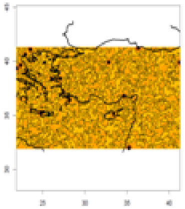 | 0.0029 | .987/.013 – .025/.975 |

**PC2**

External datasets - STR:

This analysis was aimed at testing the reproducibility of the frequency clines for the alleles which showed significant correlograms in our work (Supplemental Fig. 6).

Data of allele frequencies at the relevant loci, in populations within the geographical range here examined, were downloaded from the ALLST*R (Autosomal Database for short tandem repeats; http://allstr.de/allstr/home.seam).

National samples without detailed geographical provenance were assigned the coordinates of the capital city of that nation or region (Istanbul for Turkey). When more than one national sample was reported without detailed geographical provenance, the most numerous one was considered. A spreadsheet with the compilation of frequencies is available on request.

Frequency surface maps were obtained as described in the Materials and Methods section, with the inclusion of the Czech and Palestinian outgroups, to preserve the geographical frame. Note that not all samples were genotyped at the considered loci. So, the number of samples in each map may be different. Maps for alleles identified by our sPC 1 and 2 are drawn in red and green tones, respectively.

Results:

As opposed to our collection of locations, the external STR dataset covered mostly the Balkan peninsula. The number of samples ranged between 13 and 26, with a much greater spacing between samples. The corresponding surface maps are shown side-by-side with those of Supplemental Fig. 6.

For all alleles the frequencies in the external dataset were comparable to those of our locations.

Allele D10S1248(13) showed a cline with increasing frequencies from East to West, over the full range. The Lebanese sample displayed the second lowest frequency whereas two samples confirmed among the highest frequencies around the Austrian, Czech, Slovak border. The plot captured a spot of low frequency in Continental Greece (placed at Athens).

The focus of high frequencies of allele D10S1248(14) in Greece was fully confirmed. The area of low frequencies on the Western Greek coast seem to extend further northward. The frequency surfaces are arranged along the North-South direction.

For allele D2S1338(17) an increasing gradient from North-East to South-West was fully confirmed. High frequencies replicated in many of our locations of Calabria and Sicily were confirmed in the external dataset.

The East-to-West decreasing gradient of allele D1S1656(13) was confirmed, despite the lack of data for Northern Greece and the Aegean Islands. Romania, showing up for high frequency, was not represented in our series.

High frequencies of allele D16S539(12) North to the Balkan peninsula were confirmed by multiple points in the external dataset. The overall direction of the cline was from East to West.

The two plots for allele D18S51(19) are discrepant for the direction of the cline. We attribute this effect to the low frequencies of this allele throughout the range, which may have caused large sampling variations. Nevertheless, the relatively higher frequency in Continental Greece was confirmed in the single Greek sample (placed at Athens).

Finally, an overall increasing East-to-West cline for D3S1358(18) was confirmed, though the external datatset displayed a paucity of points in Italy and Greece, but abundant points North of the Balkan peninsula, with highs and lows.

## sPC1

**Our dataset** | **External dataset**

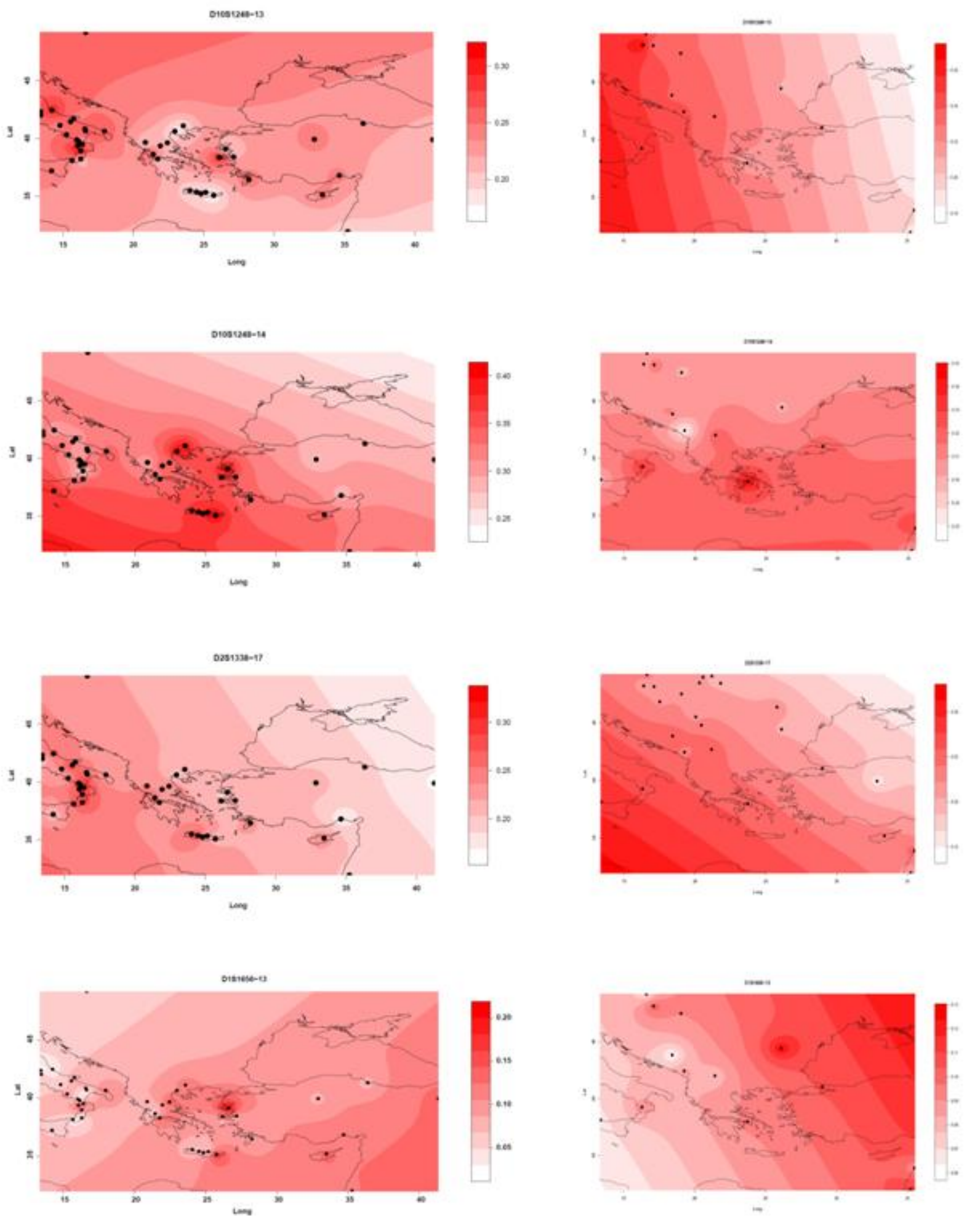

sPC2

Our dataset External dataset

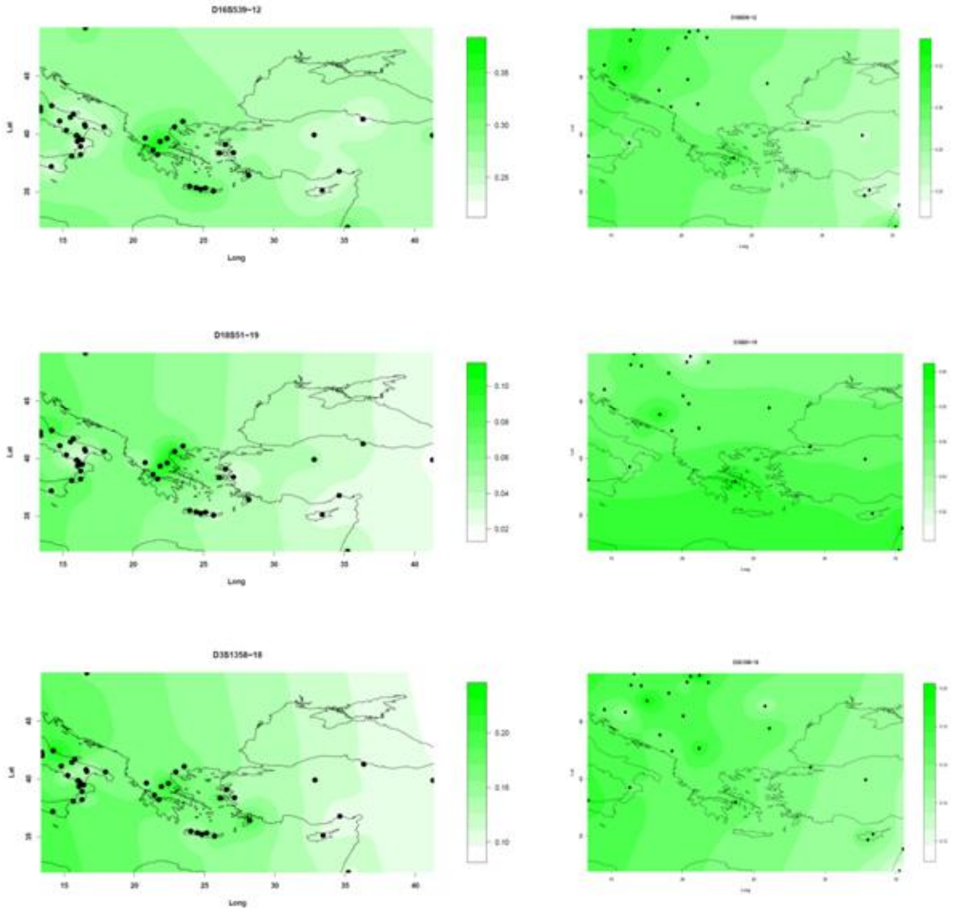

External datasets - SNP:

This analysis was aimed at verifying, over the geographical range here explored, genetic discontinuities possibly revealed by alternative markers, i.e. SNPs. We then selected the populations falling within our spatial frame from within two previously published genome-wide studies.

Data were downloaded from

http://genetics.med.harvard.edu/reichlab/Reich_Lab/Datasets.html (Lazaridis et al. 2014).

and http://www.drineas.org//Maritime_Route/RAW_DATA/ (Paschou et al. 2014)

These contain individual genotypes at 594,924 and 75,194 sites, respectively, with a variable representation of Turkey, Greece, the Aegean Islands and the Balkans. Both detasets contain Northern and Central Italians and Sicilians, whereas only one subject from Continental Southern Italy is present in the Lazaridis' dataset. Both datasets contain the CEPH Palestinians. The two datasets were analysed separately. The populations retained for the analyses are listed below.

| Lazaridis et al. | Paschou et al. |
|---|---|
| Albanian | Cappadocia |
| Bulgarian | Crete |
| Croatian | Dodecanese |
| Cypriot | East Rumelia |
| Czech | Hungary |
| Greek | Northern Italy (Bergamo) |
| Hungarian | Central Italy (Tuscan) |
| Northern Italy (Bergamo) | Macedonia |
| Central Italy (Tuscan) | Palestinian |
| Southern Italy | Peloponnese |
| Lebanese | SE_Laconia |
| Maltese | Serbia |
| Palestinian | Sicilian |
| Sicilian | |
| Syrian | |
| Turkish | |

The datasets were analysed with SMARTPCA (Patterson et al. 2006), under default settings. Plots were obtained with R.

Results:

In a first run of analyses, the CEPH Palestinians dominated the first two PCs (not shown), in agreement with both original reports.

When the Palestinians were excluded, 171 and 344 subjects remained after removal of the outliers for the Lazaridis' and Paschou's datasets, respectively. We obtained the plots reported below (comparable colour labels in the two panels). In both datasets, PC1 separated the subjects along a South-East-to-North-West axis, whereas PC2 distinguished within the Turkish group in Lazaridis'

data and separated the 10 well characterized subjects from Cappadocia (Central Turkey) in Paschou's data.

When considering only the PC1 values, two ragged distributions were obtained (histograms at bottom, with coloured points overlaid to highlight each contributing population). In both histograms, extreme positive PC1 values corresponded to populations East to the Aegean Sea, whereas negative values corresponded to populations North of the mid-Western Balkans. In between, two clusters were observed. The first grouped Northern/Central Italians with the Greeks and Bulgarian/South-eastern Balkan populations (in both datasets), whereas the second was formed mainly by Sicilians and the single Southern Italian (in Lazaridis) or Sicilians, Cretans and Dodecanese (in Paschou).

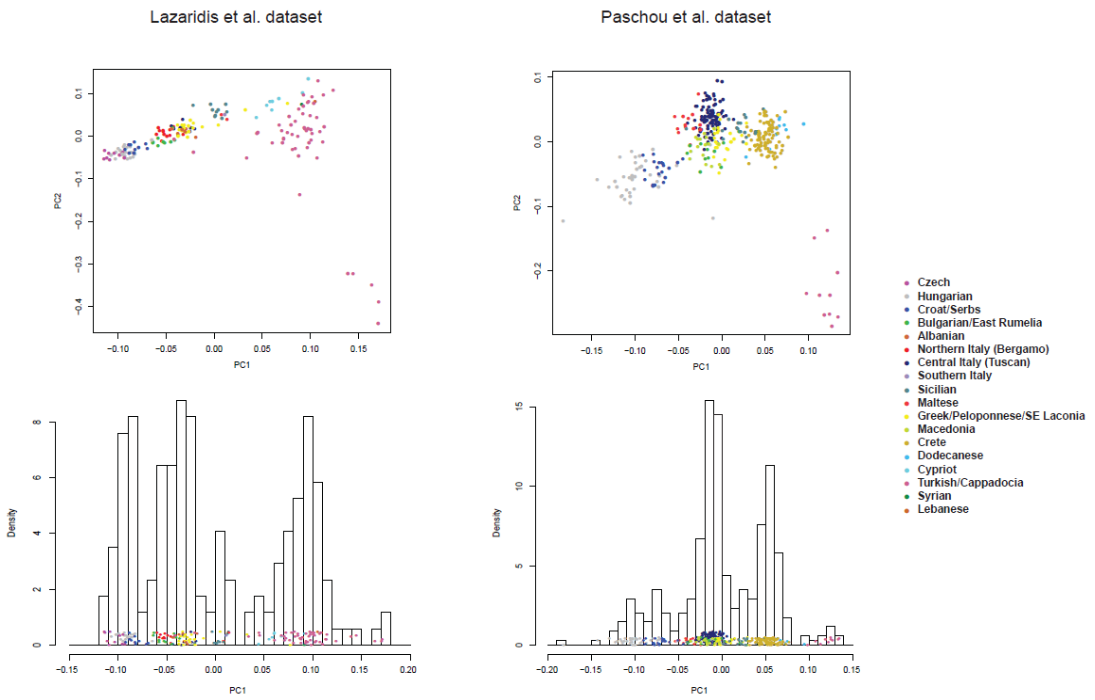

REFERENCES


Guillot G, Mortier F, Estoup A. 2005. Geneland: A computer package for landscape genetics. Mol Ecol Notes 5:708-711.

Jombart T, Devillard S, Dufour AB, Pontier D. 2008. Revealing cryptic spatial patterns in genetic variability by a new multivariate method. Heredity 101:92-103.

Lazaridis I, Patterson N, Mittnik A, Renaud G, Mallick S, Kirsanow K, Sudmant PH, Schraiber JG, Castellano S, Lipson M, et al. 2014. Ancient human genomes suggest three ancestral populations for present-day Europeans. Nature 513:409-413.

Paschou P, Drineas P, Yannaki E, Razou A, Kanaki K, Tsetsos F, Padmanabhuni SS, Michalodimitrakis M, Renda MC, Pavlovic S, et al. 2014. Maritime route of colonization of Europe. Proc Natl Acad Sci USA 111:9211-9216.

Patterson N, Price AL, Reich D. 2006. Population structure and eigenanalysis. PLoS Genet 2:e190.
